# Mindfullness-Based Interventions for Anxiety and Depression

**DOI:** 10.1192/j.eurpsy.2023.2009

**Published:** 2023-07-19

**Authors:** B. L. B. Mesquita, F. Ribeirinho Soares, M. Fraga, M. Albuquerque, J. Facucho, P. Espada, S. Paulino, P. Cintra

**Affiliations:** Department of Psychiatry, Hospital de Cascais, Lisbon, Portugal

## Abstract

**Introduction:**

Mindfulness refers to a process that leads to a mental state characterized by non-judgmental awareness of the present moment experience, including one´s sensations, thoughts, bodily states, consciousness, and the environment, while encouraging openness, curiosity, and acceptance.

**Objectives:**

The purpose of this paper is to review the ways in which cognitive and behavioural treatments for depression and anxiety have been advanced by the application of mindfulness practices.

**Methods:**

Brief non-systematic literature on the topic.

**Results:**

Mindfulness has spread rapidly in Western psychology research and practice, in large because of the success of standardized mindfulness-based interventions, consequently research on mindfulness based interventions (MBIs) has increased exponentially in the past decade. The most common include Mindfullness-Based Stress Reduction (MBSR) and Mindfulness-Based Cognitive Therapy (MBCT), which incorporate the essence of Eastern mindfulness practices into the Western cognitive-behavioral practice. MBIs have showed efficacy in reducing the severity of anxiety and depressive symptoms in a broad range of treatment seeking individuals. MBIs have also been showed to perform with not so different results to cognitive-behavioral therapy (CBT).

**Conclusions:**

MBIs have been showed to be important co-adjuvants to pharmacological treatment and psychotherapy of depression and anxiety. To prove this point without doubts and create adequate guidelines that include these forms of treatment more research needs to be done on the matter.

**Disclosure of Interest:**

None Declared

